# Clinician Emotions Surrounding Pediatric Oncology Patient Deterioration

**DOI:** 10.3389/fonc.2021.626457

**Published:** 2021-02-25

**Authors:** Dylan E. Graetz, Emily Giannars, Erica C. Kaye, Marcela Garza, Gia Ferrara, Mario Rodriguez, Dora Judith Soberanis Vasquez, Alejandra Mendez Aceituno, Federico Antillon-Klussmann, Jami S. Gattuso, Karen L. Andes, Belinda N. Mandrell, Justin N. Baker, Carlos Rodriguez-Galindo, Asya Agulnik

**Affiliations:** ^1^ Department of Global Pediatric Medicine, St. Jude Children's Research Hospital, Memphis, TN, United States; ^2^ Department of Public Health, Emory University School of Public Health, Atlanta, GA, United States; ^3^ Division of Quality of Life and Palliative Care, St. Jude Children's Research Hospital, Memphis, TN, United States; ^4^ Department of Oncology, Unidad Nacional de Oncología Pediátrica, Guatemala City, Guatemala; ^5^ Department of Nursing, Unidad Nacional de Oncología Pediátrica, Guatemala City, Guatemala; ^6^ Department of Critical Care, Unidad Nacional de Oncología Pediátrica, Guatemala City, Guatemala; ^7^ Unidad Nacional de Oncología Pediátrica, Guatemala City, Guatemala; ^8^ Francisco Marroquin University School of Medicine, Guatemala City, Guatemala; ^9^ Department of Nursing Research, St. Jude Children's Research Hospital, Memphis, TN, United States

**Keywords:** cancer, critical care, pediatric oncology, early warning system, qualitative research

## Abstract

**Background:**

Pediatric oncology patients have a high rate of clinical deterioration frequently requiring critical care. Patient deterioration events are distressing for clinicians, but little is known about how Pediatric Early Warning Systems (PEWS) impact clinicians’ emotional responses to deterioration events.

**Methods:**

Semi-structured interviews were conducted with 83 nurses, pediatricians, oncologists, and intensive care clinicians who had recently participated in a patient deterioration event at two pediatric oncology hospitals of different resource-levels: St. Jude Children’s Research Hospital (N = 42 participants) in Memphis, Tennessee or Unidad Nacional de Oncología Pediátrica (N = 41 participants) in Guatemala City, Guatemala. Interviews were conducted in the participants’ native language (English or Spanish), transcribed, and translated into English. Each transcript was coded by two researchers and analyzed for thematic content.

**Results:**

Emotions around patient deterioration including concern, fear, and frustration were reported across all disciplines at both hospitals. Concern was often triggered by an elevated PEWS score and usually resulted in increased attention, which reassured bedside clinicians that patients were receiving necessary interventions. However, persistently elevated PEWS scores, particularly at St. Jude Children’s Research Hospital, occasionally resulted in a false sense of relief, diminishing clinician attention and negatively impacting patient care. Nurses at both institutions described how PEWS amplified their voices, engendering confidence and empowerment, two of the only positive emotions described in the study.

**Conclusion:**

Clinicians experienced a range of emotions while caring for high-risk patients in the setting of clinical deterioration. These emotions have the potential to contribute to compassion fatigue and burnout, or to resilience. Acknowledgment and further investigation of the complex interplay between PEWS and clinician emotions are necessary to maximize the impact of PEWS on patient safety while simultaneously supporting staff wellbeing.

## Introduction

Hospitalized children who deteriorate often show signs and symptoms of clinical decline in the 24 h preceding decompensation (1, 2). Early detection strategies, such as Pediatric Early Warning Systems (PEWS), are instrumental in preparing for and preventing rapid deterioration on the ward (1). PEWS are nursing-administered clinical acuity tools associated with an escalation algorithm to facilitate early detection of children at-risk of decompensation. Pediatric oncology patients have a high rate of clinical deterioration due to disease burden and treatment toxicity, and approximately 40% will require admission to the pediatric intensive care unit (PICU) during the course of their cancer-directed therapy (3). Thus, PEWS are particularly important for this population (4, 5) and were recently identified as a top research priority to improve care of critically ill pediatric oncology patients (6).

While PEWS have been primarily implemented and studied in high-resource settings, pediatric cancer is a global health issue, with approximately 90% of pediatric cancer diagnoses occurring in countries with limited resources (7). Previous studies have shown that PEWS implementation in a resource-limited pediatric oncology setting reduces clinical deterioration events and hospital costs, while improving interdisciplinary communication (5, 8, 9).

In addition to impacting patients, clinical deterioration events are stressful to bedside clinicians. Clinician stress and lack of emotional resilience contribute to burnout (10, 11) and may affect clinical judgment and decision-making, essential to the evaluation and care of sick children (12, 13). Limited research exists on how clinicians’ emotions affect patient care and how interventions like PEWS impact these emotions or modify patient safety. It is also poorly understood whether clinician stress and emotions around patient deterioration vary by hospital resource-level. To address this deficit, we qualitatively investigated the relationship between PEWS and pediatric oncology clinicians’ emotions surrounding deterioration events in two hospitals of different resource levels, with the goal of informing PEWS implementation and global patient safety improvement efforts and improving clinician experiences.

## Methodology

Multidisciplinary pediatric oncology clinicians were interviewed at Unidad Nacional de Oncología Pediátrica (UNOP) and St. Jude Children’s Research Hospital (SJCRH), two free-standing pediatric oncology hospitals, between October and December 2018. Detailed methods of this study including setting, population, and data collection have been previously described (8). Briefly, UNOP is located in Guatemala City, Guatemala, an upper middle-income country with a gross national income (GNI) of US $4,410 per capita. UNOP is considered a low-resource setting, characterized by insufficient supplies and staffing. There is approximately one oncologist for every 66 newly diagnosed patients per year at UNOP, and four intensivists for a nine-bed pediatric intensive care unit (PICU) with 300–400 admissions per year. Nurse to patient ratios are 1:4–1:6 on inpatient wards and 1:1–1:2 in the PICU. SJCRH is located in Memphis, TN, USA with a GNI of US $62,850 per capita and is considered a high-resource setting. SJCRH has approximately one oncologist for <15 newly diagnosed oncology patients per year and eight intensivists for a 12-bed PICU with 350–400 admissions annually. Nursing ratios in the inpatient wards are 1:2 and in the PICU are 1:1.These hospitals were selected for the study based on their similar missions, patient populations, patient volume, and processes of PEWS implementation. PEWS, or “EVAT”, was implemented at UNOP in 2014 (4) and at St. Jude, as “SJAWS”, in 2016 (14). Key stakeholders, including ICU physicians, fellows, ward physicians, advance practice practitioners, bedside nurses, and nursing managers, were involved in all stages of implementation at both sites. During implementation, each hospital conducted weekly monitoring of PEWS documentation by evaluating errors. Both hospitals were able to achieve similarly low error rates during implementation and have maintained these rates through ongoing quality monitoring and re-education (5, 14). PEWS algorithms and scoring tools are similar at each site and are included as **Supporting Material**.

Participants from UNOP and SJCRH included multidisciplinary staff involved in care escalation, including bedside nurses and unit nursing coordinators, frontline physicians (pediatricians and pediatric hematology-oncology fellows), and critical care clinicians, such as attending physicians and fellows, advanced practice practitioners (SJCRH only), and critical care nursing coordinators (SJCRH only). Participants were selected based on their involvement in a recent deterioration event, defined as an unplanned transfer to the PICU.

Interviews were conducted in the participant’s native language and simultaneously transcribed and translated into English as necessary. Transcribed interviews underwent analysis utilizing inductively derived themes (15). Further thematic analysis was conducted to identify emotional patterns across the three disciplines and two hospital sites. Segments coded as “emotions” were explored, focusing specifically on overlap between “emotions” and “negative perception [of deterioration event]” and “positive perception [of deterioration event]”. MAXQDA software (VERBI GMBH, Berlin, Germany) was used for data management and analysis. Consolidated criteria for reporting qualitative research (COREQ) guidelines (16) were followed to ensure quality in qualitative reporting.

Study personnel who approached potential participants were not involved in PEWS implementation or the discussed deterioration events. Verbal consent was obtained from participants in English or Spanish; the study was explained in participants’ native language by a native speaker. No identifying information was collected from participants and participants were asked to avoid using private health information during the interview. This study was exempt from SJCRH Institutional Review Board (IRB) approval and approved by UNOP IRB.

## Results

A total of 83 interviews were conducted; 42 were conducted at SJCRH and 41 at UNOP. **Table 1** describes participant demographics at both institutions. Analysis revealed three main themes, including “negative clinician emotions around patient deterioration and care escalation,” “impact of PEWS on emotions,” and “confidence and clinical judgment.”

**Table 1:** Demographics of Interviewed Participants.

Health Care Provider	SJCRH n (%)	UNOP n (%)
Nurses	13 (31)	20 (49)
Coordinator	2 (5)	8 (20)
Bedside Nurse	11 (26)	12 (29)
Ward physicians	16 (38)	14 (34)
Oncology fellow	6 (14)	6 (14)
Resident/pediatrician	3 (7)	8 (20)
APP (NP, PA)	7 (17)	N/A
PICU provider	13 (31)	7 (17)
PICU nurse	2 (5)	N/A
APP (NP, PA)	5 (12)	N/A
PICU fellow	N/A	6 (15)
PICU attending physician	6 (14)	1 (2)
**Total**	42 (100)	41 (100)

APP, advanced practice provider; N/A, not available; NP, nurse practitioner; PA, physician assistant; PICU, pediatric intensive care unit; SJCRH, St. Jude Children’s Research Hospital; UNOP, Unidad Nacional de Oncología Pediátrica.

### Negative Clinician Emotions Around Patient Deterioration and Care Escalation

Patient deterioration events stimulated feelings of concern, worry, frustration, stress, fear, lack of confidence, and regret across participants at both institutions (**Table 2**). At UNOP, a few clinicians mentioned feelings of anger related to patient deterioration: *“…I got angry because he died in my care. I got him, I intubated him, I talked to the parents, the parents were desperate, he could not handle it, it was terrible”* (PICU clinician, UNOP). Anger was not explicitly described by clinicians at SJCRH.

**Table 2:** Provider emotions around patient deterioration.

	SJCRH	UNOP
**Concern/Worry**	*“I actually stood in the room with the nurse because she was so concerned, and I stood there, and I remember it taking so long…” (ward provider)*	*“Something like a neurological matter, I will be worried if the nurse says: ‘the boy has difficulties to speak’ or ‘he can’t move his legs’ or if I find leukoencephalopathy or neurological deterioration, bleeding, it’s time to act!” (ward provider)*
**Frustration**	*“it was very frustrating because he would kind of arouse with all of the people in the room, but he still wasn’t himself.” (ward provider)* *“So, it’s frustrating when you know, the Mom looks at us, why didn’t you send this earlier?” (ward provider)*	*“the only thing that exasperated me was that they didn’t transferred the patient faster, due to all the movements needing to be done in intensive care, so it is difficult, as some times intensive care does not have the space when you needed it, and sometimes takes them time to clean the beds and during that time the patient gets worse over here.”(nurse)*
**Stress**	*“The PICU felt like they were kind of being – they were kind of managing that patient anyway, and so they might as well go ahead and move him over and remove that stress from the [ward] team.” (ward provider)*	*“I think that with stress they are thinking about what to do and what not to do, like they are doubting what they should do and what they shouldn’t do.” (nurse)*
**Fear**	*“Our fear is that something will be missed and some number will change and something will fall through the cracks.” (ward provider)*	*“…because sometimes nursing, when a child has been in intensive a lot, they get more scared, they don’t like it and I understand why, in the service they want someone else to handle the most complex child…” (PICU provider)* *“That is what we fear being up here and we also have many patients for just one person.” (nurse)*
**Lack of confidence**	*“I do believe a lot of times we monitor our patients a little bit longer than we probably should have before just because of a lack of confidence.” (nurse)*	*“The problem is that I feel they lack more confidence in using it, because they often ask the nurse’s coordinator if the [PEWS] is well-placed or not.” (nurse)*
**Regret**	*“With a lot of hindsight, you can see where things have been missed and, regrettably, things that could’ve been done differently mostly likely in some situations.” (PICU provider)*	*“That day the patient was transferred to intensive care at 8am…, I complied with everything … everything was done. but you always go thinking … maybe I should have done this. there are always three points that I need to work on, right?”(nurse)*

Concern was the emotion mentioned most frequently by clinicians. Increased concern in response to patient deterioration was mentioned across all disciplines at both hospital sites, but particularly by nurses, *“We are concerned that the patient is hypotensive … we review the capillary refill … [check] if the patient is reacting normally, because if he is not, that also worries us”* (nurse, UNOP). Clinicians also worried if the patient’s status changed from baseline: *“…the progression of his symptoms, that’s what worried me, not the specific number”* (PICU physician, SJCRH).

If clinician concern was met with a perceived lack of action (*i.e.*, PICU consult, care escalation), it fostered more negative feelings. At SJCRH, nurses became frustrated when they felt like their worries weren’t being received appropriately by others*: “Once I actually know that it’s a problem and they’re not really doing anything about it, that’s when I have more concerns … when I send my patients to the PICU, I was like we need to do something other than keep telling you every two hours, hey we’re still at this number”* (nurse, SJCRH). SJCRH PICU clinicians also expressed frustration when appropriate action was not taken by the ward team: *“When they continue to call you and not take your recommendations, that’s frustrating”* (PICU clinician, SJCRH).

UNOP nurses’ worries were similarly amplified when they were unable to act or escalate care. As at SJCRH, nurses at UNOP sometimes felt ignored by physicians: “*Sometimes they do not give as much importance to the [PEWS] as us, for example, they say ‘no, he is not that bad’”* (nurse, UNOP). However, lack of action at UNOP was also noted as the result of limited essential resources: *“We are worried about having a complicated patient here because we do not have the equipment, we do not have for example a defibrillator”* (nurse, UNOP).

Conversely, when care was appropriately escalated it eased the burden of taking care of deteriorating patients on the ward for both UNOP and SJCRH clinicians: *“You feel more at ease when you know that a patient is already at intensive care. The personnel down there know how to manage these types of patients and they know what needs to be done…”* (nurse, UNOP). Similar perspectives were shared by physicians: *“There’s definitely a lot more patients that have been going to the PICU for observation for 24 to 48 hours, which I think is an improvement over previously, when previously we were managing these patients on the floor. There was a lot of distress over taking care of and worrying about”* (ward physician, SJCRH).

Clinicians across disciplines at both institutions expressed frustration surrounding deterioration events; *“we become inaccessible … we get annoyed”* (PICU clinician, UNOP); “*…we know like certain attendings are more likely to take a patient faster than others … it’s not going to change, but it was very frustrating”* (ward clinician, SJCRH). In addition, ward clinicians at SJCRH and nurses at UNOP expressed feeling stress or fear when caring for deteriorating patients: *“So, having three patients like this with the altered [PEWS] causes a little stress, then … the communication starts to fail”* (nurse, UNOP). Nurses at both institutions described feeling intimidated or lacking confidence. This phenomenon was mentioned by other clinicians as well, who acknowledged how their actions might at times evoke nursing apprehension: “*But I think also maybe we make them a little nervous so they’re afraid to really say what they’re worried about … some of us may be a little harsher and maybe say things that have made them feel bad and so then they don’t want to speak up…”* (PICU clinician, SJCRH). As clinicians reflected on deterioration events, some expressed remorse about things that could have gone differently: *“If something were to happen to them and we didn’t take that extra step, then we will regret that”* (ward clinician, SJCRH).

### Impact of PEWS on Emotions

**Figure 1:**
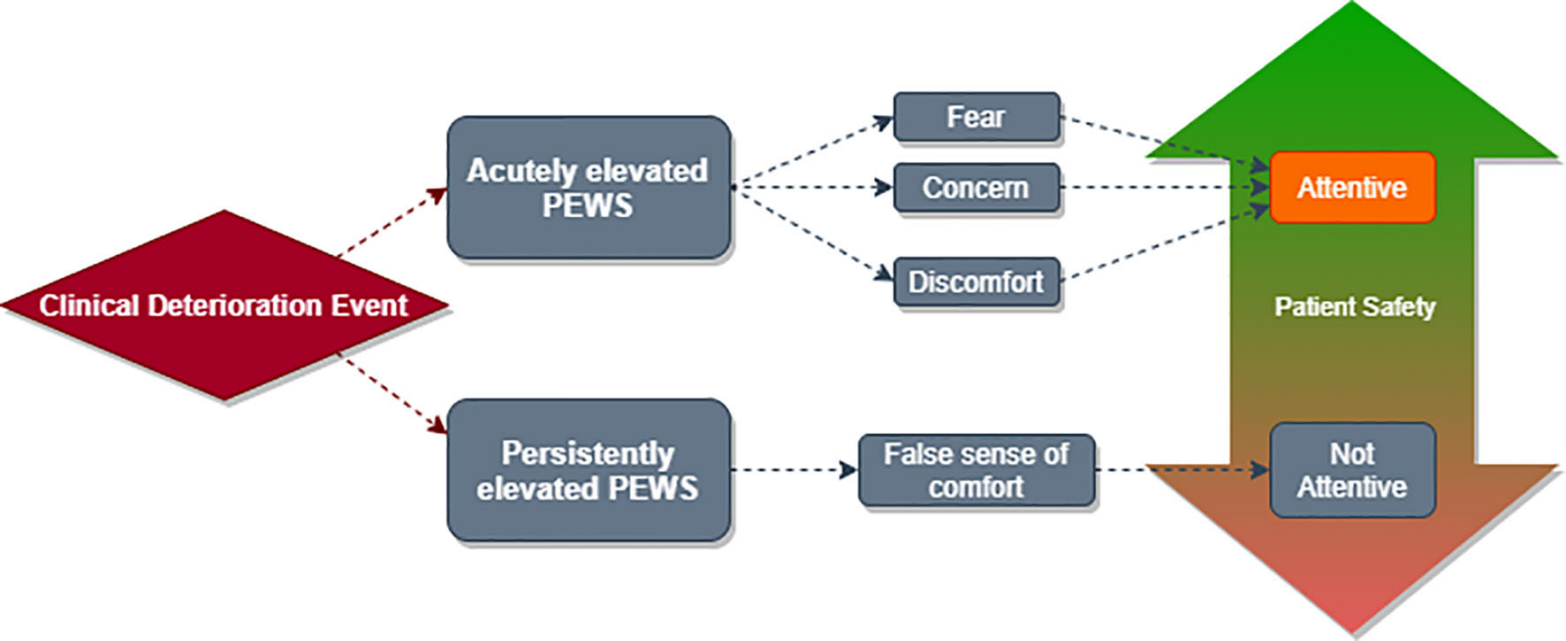
Interaction between clinical deterioration events, PEWS scores, provider emotions, and patient safety. Clinical deterioration events result in elevated PEWS scores. If a PEWS score is elevated acutely, as it may be at the beginning of a deterioration event, clinicians experience emotions such as fear, concern, and discomfort. These emotions may be negative as experienced and described by clinicians; however they often lead to increased patient attention and safety. Conversely, a persistently elevated PEWS score may lead clinicians to experience a false sense of comfort which results in decreased attention and reduced patient safety.

Depending on the situation, PEWS either heightened or relieved clinicians’ negative emotions around deterioration events, which subsequently affected clinician attention and response (**Figure 1** **;** **Table 3**). In many cases of clinical deterioration, PEWS increased concern among clinicians by acting as a warning signal that a patient’s status was declining: “*If a nurse gets a high [PEWS] and then call me … it will make the nurse more aware [and] makes me as the physician more aware”* (ward clinician, SJCRH). While an uncomfortable emotion, this concern encouraged clinicians to be more alert and attentive to their patients: *“When they already have a [PEWS] number 3… we monitor the patient, we are already concerned and checking on him every hour to think about a transfer to another service”* (nurse, UNOP).

**Table 3:** Impact of PEWS on provider emotions.

	SJCRH	UNOP
**PEWS scores alerting providers and encouraging action**	*“I think that’s built into the structure with the recognition that the [PEWS] is to identify those patients who are risk of decompensating that we’re worried about” (ward provider, SJCRH)* *“I feel like if they’re repeatedly a [PEWS] of a five there’s something wrong” (nurse, SJCRH)*	*“For us, it is worrying to have a [PEWS] of 4, 5, or 6” (nurse, UNOP)* *“When I see the [PEWS] report and the nurse shows worries, I call her” (ward provider, UNOP)* *“What I really look [at] is the [PEWS]. If it’s a 3, the child has tachycardia or something [else] is wrong … at that moment I check the vitals to look closer” (ward provider, UNOP)*
**PEWS scores contributing to alarm fatigue**	*“PICU people may be getting a little more burnout from having to do so many consults with the [PEWS] of five” (nurse, SJCRH)* *“I feel poorly when I go to a [PEWS] and I’m kind of like, yeah the kid looks fine” (PICU provider, SJCRH)*	

The PEWS result itself also created a sense of urgency. If a patient had a higher score, clinicians worried about them more than a patient with a lower score. This was expressed by PICU clinicians at both hospitals: *“A seven or eight, I’m going to have a very different feeling than a five. I’m going to feel a little more urgency to get to the room”* (PICU clinician, SJCRH); *“I think one is more alarmed when the* [PEWS] *is higher, and I think part of being alarmed a little more is paying more attention. For example, he has* [a PEWS] *of five, let’s better review him”* (PICU clinician, UNOP). Alternatively, the absence of an elevated PEWS score brought a sense of comfort that the patient was at a low risk of deterioration: *“If we see a* [PEWS] *of zero, I presume everything is fine”* (ward clinician, UNOP).

When a patient had a consistently elevated PEWS score, emotions were mixed. For some clinicians, persistently elevated PEWS induced a sense of foreboding: *“I feel like if they’re repeatedly* [a PEWS] *of a five there’s something wrong”* (nurse, SJCRH). On the other hand, at SJCRH, it was frequently mentioned that persistently elevated scores manifested as alarm fatigue, resulting in a false sense of security among clinicians. SJCRH PICU clinicians expressed that repeat elevated scores lessened the value of the PEWS and caused some clinicians to ignore the calls: *“I think that’s where we’ve seen failures in the system, where a kid always has* [a PEWS] *of five or six or whatever and then we just ignore them”* (PICU clinician, SJCRH). Ward clinicians endorsed similar concerns about alarm fatigue, admitting that it did lead to frustration and ignoring calls: *“…someone has an [PEWS score] at all, I feel like we should be concerned about it, but we just hear they have this number so often that it almost like means nothing to us at this point”* (ward clinician, SJCRH); *“How do you make it so that we don’t develop alarm fatigue when … they’re always* [PEWS] *three … I’m not concerned and then start to ignore these things or kind of get frustrated with the fact that every couple of hours I’m getting paged or called”* (ward physician, SJCRH). Even nurses, as direct bedside clinicians, endorsed feeling a false sense of security with persistently elevated scores: “*Kids that look unwell but are maintaining a high* [PEWS] *score … I think it also makes us nurses slack a little bit too, you’re like, oh they’re just a four, and you’re like, we get like, comfortable with a four or a five when we, those vitals are not normal”* (nurse, SJCRH). Participants from all disciplines at SJCRH mentioned this phenomenon. While participants at UNOP discussed negative elements of PEWS, including “false positives” or PEWS creating increased concern around well children, alarm fatigue did not appear in any transcripts from UNOP.

### Confidence and Clinical Judgment

One of the only positive emotions around deterioration events expressed by clinicians at both institutions was confidence. This emotion was endorsed by clinicians from both sites who expressed that PEWS increased their confidence by providing evidence to substantiate their clinical acumen or assessment that a patient was deteriorating: *“…it does give us a little more concrete evidence to base our feelings on and our perceptions of how patients do … it makes us more comfortable reaching out to a physician or nurse practitioner saying that we’re concerned if we have evidence to back it up”* (nurse, SJCRH);*”We give our own criteria of what we think is happening and how it can be resolved … now it is more valued what nursing requests, or what nursing reports”* (nurse, UNOP). Advance practice providers and physicians working on the ward and in the PICU at both institutions noted this confidence: *“I think that it has certainly given the nurses more latitude to call for medical intervention earlier on. Especially I think for newer nurses or younger nurses. It’s easier than just saying, ‘Well, I don’t know why but I’m just worried about this patient.’ They can actually have a more sort of concrete, ‘I had to call you because the [PEWS] is blank’”* (ward clinician, SJCRH). **Table 4** includes quotes from clinicians in all disciplines at both institutions illustrating how PEWS increased nursing confidence and facilitated interdisciplinary communication around concerns regarding patient deterioration.

**Table 4:** Nursing confidence inspired by PEWS.

SJCRH	UNOP
*“[PEWS gives] the ability to have the nurses feel comfortable that*—*now they don’t have to just keep asking for help if they need something or if there’s a concern, [PEWS] easily prompts us.” (PICU provider)*	*“Here there is much more openness on the part of the intensive to people from outside. In other places they are afraid of the intensive, of going to ask, because they think that they are going to shout or call them fools.” (PICU provider)*
*“If the nurse is ever scared, we’re getting called.” (PICU provider)*	*“I trust because the nurses are our “eyes” our “hands” we can’t do everything ourselves so we must trust on the nurse.” (ward provider)*
*“I think that it’s great for the nurses because it gives them the ability to not feel bad [calling] a rapid response.” (ward provider)*	*“I go to find them and tell my worries because the signs are fine but there is something that I don’t like … a lot of pain or so … I go to look for them in person, I prefer that way.” (nurse)*
*“I think it gives the nurse an opportunity to empower themselves to not feel judged, they’re basically to be taken seriously.” (ward provider)*	*“Here if there is something that the oncologists have given us confidence is that they have always taken us into account.” (nurse)*
*“I like [PEWS], I think it has really made me be more aware of my patient and like the subtle changes, and I feel more comfortable calling an [rapid response]now, and I think at first when it first started the doctors were a little hesitant, like when we kept calling, like they’re at a four or something like that, but now it’s like very open communication.” (nurse)*	*“Because if we worry about him, it is because we know the patients, we are here with them and we are the ones who share the most with them.” (nurse)*
*“Because of the [PEWS] you can confidently contact PICU, and not be worried there’s going to be backlash or whatever.” (nurse)*	*“Then I feel sure that the patient is fine, and I will go with the doctor to tell him that he’s okay, so I go with the assurance that I did the [PEWS] well and that I evaluated the patient well.” (nurse)*

## Discussion

Pediatric oncology clinicians work in emotionally-charged environments. Emotions have been shown to affect clinical judgment and clinician response to clinical situations (17, 18), yet the relationship between emotions and patient safety is not well understood. The relationship between PEWS and patient safety, however, has been well described—PEWS facilitate improvements in patient safety by warning clinicians of concerning patient changes, prompting them to consider the possibility of deterioration and potential care escalation (19, 20). Our study reinforces these findings and presents qualitative evidence examining how PEWS interacts with clinicians’ emotions to improve patient safety.

Most of the emotions related to deterioration described by participants in this study are negative. While it is unsurprising that clinicians carry negative emotions surrounding traumatic patient deterioration events, this finding is critical in our recognition of the profound emotional toll that deteriorating patients have on clinicians. This emotional toll contributes to increased moral distress, clinician burnout and higher staff turnover in both high- and limited-resource settings (11, 21). The fact that participants across all disciplines at both hospitals discussed negative emotions also demonstrates how widespread and universal these responses are in the context of patient deterioration and critical illness.

Clinicians in this study discussed how PEWS can facilitate action and intervention, not only increasing patient safety but also providing reassurance to clinicians. The ability of PEWS to empower bedside clinicians (8) contributes to increased nursing confidence, one of the only positive emotions consistently expressed by participants in this study. Nursing confidence is essential to the provision of optimal patient care, as it promotes earlier engagement between interdisciplinary team members and consideration of care escalation. In addition, nurses who feel heard and supported may experience less moral distress (22). Confidence mitigates clinician burnout by helping clinicians feel enabled to appropriately care for deteriorating patients (23).

Nevertheless, the relationship between PEWS, clinicians’ emotions, and patient safety is complicated. While PEWS may help clinicians, and particularly nurses, feel more confident and empowered, it can also increase concern and anxiety if a clinician feels the escalation pathway is not appropriately followed. Clinicians from SJCRH specifically expressed concern about “alarm fatigue,” or a false sense of security, created by persistently elevated scores. Alarm fatigue is an important concept for ongoing discussion and evaluation, as a potential threat to patient safety (24) and a pathway towards increased clinician burnout and decreased resilience (25). The lack of alarm fatigue in UNOP transcripts is interesting and warrants further exploration. An equivalent for the term “alarm fatigue” does not exist in Spanish. However, when reflecting on PEWS and clinical deterioration events, clinicians at UNOP did not describe an inappropriate sense of security, or decreased attention to patient care due to frequent notifications. It is possible that a lack of available resources for successful care escalation may increase anxiety among nurses, leading them to be more vigilant and advocate for higher care even in the settings of repeatedly elevated scores; however, more research is needed to examine drivers of this finding.

This study has several limitations. Though PEWS implementation processes were similar at UNOP and SJCRH, specific escalation algorithms varied slightly between the sites. While our methodology allowed us to compare clinician emotions across different institutions, the culture and context of each oncology ward, along with clinician education and training, may contribute to site-specific differences in data. Social desirability bias (26) should be considered in all qualitative studies; this bias was mitigated by using external and/or clinically uninvolved interviewers and study personnel, and the identification of numerous negative emotions suggests successful limitation of this bias. Finally, this study utilized both English and Spanish interviews, with all transcripts analyzed in English. While this approach likely increased consistency across analyses, it may have influenced the intent of original statements. All transcripts were retained and reviewed as necessary, with a bilingual team member auditing 20% of Spanish language transcripts to minimize errors and inaccuracies.

Qualitative methodology allowed us to deeply investigate clinician experiences and the impact of PEWS as a quality improvement intervention. Ultimately, our findings reveal similarities between emotional responses to patient deterioration across clinicians from various disciplines at two hospitals with divergent resource levels. Although this study targeted analysis of pediatric oncology clinician experiences surrounding deterioration events, these findings may be relevant to a broader population of clinicians who care for children at high risk of critical illness. Within pediatric cancer, these data will inform a framework for developing future interventions aimed at improving clinician experiences and supporting resilience while decreasing clinician burnout in high and limited-resource settings.

## Data Availability Statement

The raw data supporting the conclusions of this article will be made available by the authors, without undue reservation.

## Ethics Statement

The studies involving human participants were reviewed and approved by St. Jude Children’s Research Hospital and Unidad Nacional Oncologia Pediatrica. Written informed consent for participation was not required for this study in accordance with the national legislation and the institutional requirements.

## Author Contributions

Study was conceptualized and designed by DG and AA. Data collection was facilitated and completed by MG, MR, DS, AMA, and JSG. Data analysis was completed by DG, EG, MG, GF, and AA. DG and EG were responsible for drafting initial manuscript. All authors were involved in data interpretation, manuscript review, and approval of final manuscript. All authors contributed to the article and approved the submitted version.

## Funding

This study was funded by the American Lebanese Syrian Associated Charities.

## Conflict of Interest

The authors declare that the research was conducted in the absence of any commercial or financial relationships that could be construed as a potential conflict of interest.
