# Supplementary material for: Clinician Emotions Surrounding Pediatric Oncology Patient Deterioration
Source: Front Oncol. 2021 Feb 25;11:626457. doi: 10.3389/fonc.2021.626457 (PMC7947818; doi:10.3389/fonc.2021.626457)
Supplement: Supplementary file 1 [file DataSheet_1.docx]

**Clinician Emotions Surrounding Pediatric Oncology Patient Deterioration**

**Supporting Material Table of Contents**

| **Item** | **Page** |
| --- | --- |
| **Supporting Figure 1: Escala de Valoración de Alerta Temprana (EVAT) PEWS Reference Tool**. | **2** |
| **Supporting Figure 2**. **Escala de Valoración de Alerta Temprana (EVAT) PEWS Escalación Algorithm**. | **3** |
| **Supporting Figure 3. St. Jude Advanced Warming Score (SJAW) PEWS Reference Tool**. | **4** |
| **Supporting Figure 4**. **St. Jude Advanced Warning Score (SJAW) PEWS Escalation Algorithm**. | **5** |


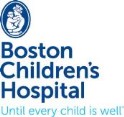
PEWS reference tool from Unidad Nacional de Oncología Pediátrica (UNOP), translated from Spanish. Reproduced with permission from Agulnik A, Mora Robles LN, Forbes PW, et al. Improved outcomes after successful implementation of a pediatric early warning system (PEWS) in a resource-limited pediatric oncology hospital. *Cancer.* Apr 25 2017.


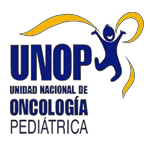


**Escala de Valoración de Alerta Temprana (EVAT)**

**Reference Tool (Translated from Spanish)**

| **Escala de Valoración de Alerta Temprana (EVAT)** | | | | | |
| --- | --- | --- | --- | --- | --- |
|  | **0** | **1** | **2** | **3** | **Result** |
| **Behavior / Neurologic** | ° Alert/Sleeping appropriately  ° Patient is at baseline state of alertness | ° Sleepy, drowsy when not stimulated  ° Responds only to verbal stimuli | ° Irritable, difficult to console  ° Responds only to painful stimuli | ° Lethargic, confused, without strength  ° Unresponsive  ° Seizures  ° Unreactive pupils or with anisocoria |  |
| **Cardiovascular** | ° Appropriate skin color for patient  ° Capillary refill ≤ 2  seconds  ° Normal peripheral pulses | ° Pale  ° Vasodilated  ° Capillary refill 3-4 seconds  ° Mild tachycardia* | ° Capillary refill 4-5 seconds  ° Moderate Tachycardia*  ° Diminished peripheral pulses | ° Mottled  ° Fill capillary> 5 seconds  ° Severe tachycardia*  ° Symptomatic bradycardia  ° Irregular rhythm (not sinus) |  |
| **Respiratory** | ° Within normal parameters  ° No retractions  ° Normal breathing pattern  ° Saturation >95% | ° Mild tachypnea*  ° Mild work of breathing (nasal flaring, intercostal retraction)  ° Up to 1 L of oxygen via nasal cannula (NC)  ° Saturation 90% -94% without oxygen | ° Moderate tachypnea*  ° Moderate work of breathing (nasal flaring, intercostal retraction, grunting, use of accessory muscles)  ° 1-3 L of oxygen via NC  ° Nebulization every 4 hrs  ° Saturation 88-89% without oxygen | ° Severe tachypnea*  ° Respiratory rate below normal for age*  ° Severe work of breathing (head-bobbing, thoraco-abdominal dissociation)  ° Oxygen via facemask with reservoir (not post-sop)  ° > 3 L oxygen via NC  ° Nebulization > every 4 hours  ° Saturation <90% with oxygen  ° Apnea |  |
| **Nurse concern** | Not concerned | Concerned |  |  |  |
| **Family concern** | Not concerned and present | Concerned or absent |  |  |  |
| **TOTAL** | | | | |  |

*** Please refer to Heart Rate and Respiratory Reference Tool**

Based on Bonafide C, et al. Development of Heart and Respiratory Rate Percentile Curves for Hospitalized Children. Pediatrics 2013;131;e1150.

|  | **Mild** | **Moderate** | **Severe** |
| --- | --- | --- | --- |
| **Respiratory rate and heart rate** | 90-95th percentile for age | 95-99th percentile for age | > 99th percentile for age |


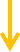


**3-4**

(Yellow)

***Managed by Hospitalist Consider:***

***Intensive Care***

***Consultation***

***Additional evaluation criteria:***

**Patients who require more than 2 boluses in previous 4 hours**

**Increased pain that exceeds the clinical condition of the patient**

- **Monitor vital signs every hour**
- **Notify the nurse coordinator and on-call physician**
- **Discuss the patient's status as a group**
- **Consider a higher level of care**
- **Document interventions**


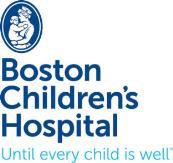
PEWS escalation algorithm from Unidad Nacional de Oncología Pediátrica (UNOP), translated from Spanish. Reproduced with permission from Agulnik A, Mora Robles LN, Forbes PW, et al. Improved outcomes after successful implementation of a pediatric early warning system (PEWS) in a resource-limited pediatric oncology hospital. *Cancer.* Apr 25 2017.


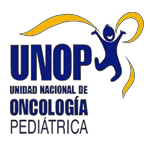


**EVAT**

**Escala de Valoración de Alerta Temprana**

Escalation Algorithm (Translated from Spanish)

**0-2**

(Green)

**Continue routine care**

**5 o >**

(Red)


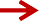


***Additional evaluation criteria:***

- **Patients who require continuous nursing care for> 1 hour**
- **Presence of systolic or diastolic hypotension**
- **Clinical physician evaluation at patient’s bedside**
- **Notify attending oncologist**
- **Discuss the patient's status as a group**
- **Continuous monitoring**
- **Document interventions**

***Mandatory Intensive Care Consultation***

For immediate assistance at any time:

**CALL the PICU: 255**

PEWS reference tool from St. Jude Children’s Research Hospital (St. Jude). Reproduced with permission from Agulnik, A. Forbes, P. Kleinman, M. Rodriguez-Galindo, C., & Stenquist, N. (2016). Validation of a pediatric early warning score in hospitalized pediatric oncology and hematopoetic stem cell transplant patients. *Pediatric Critical Care Medicine,* 17(4). e146-153


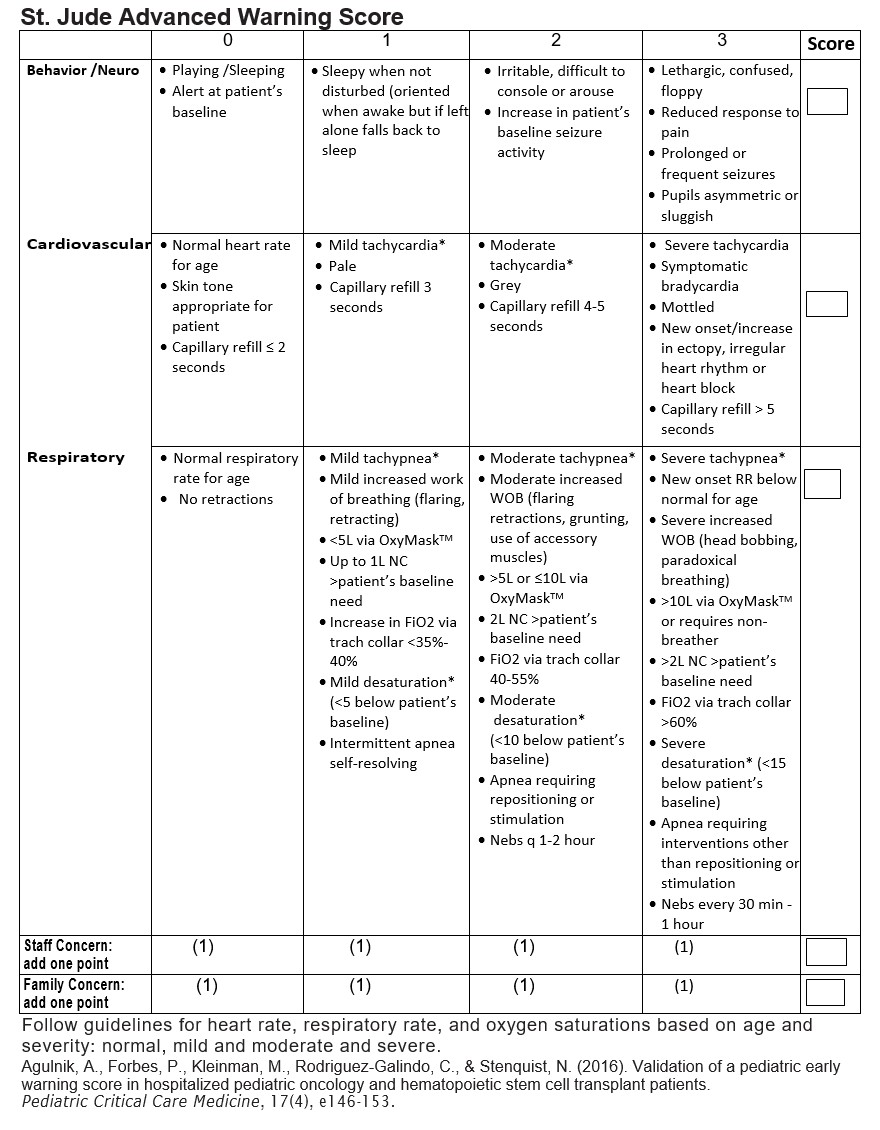


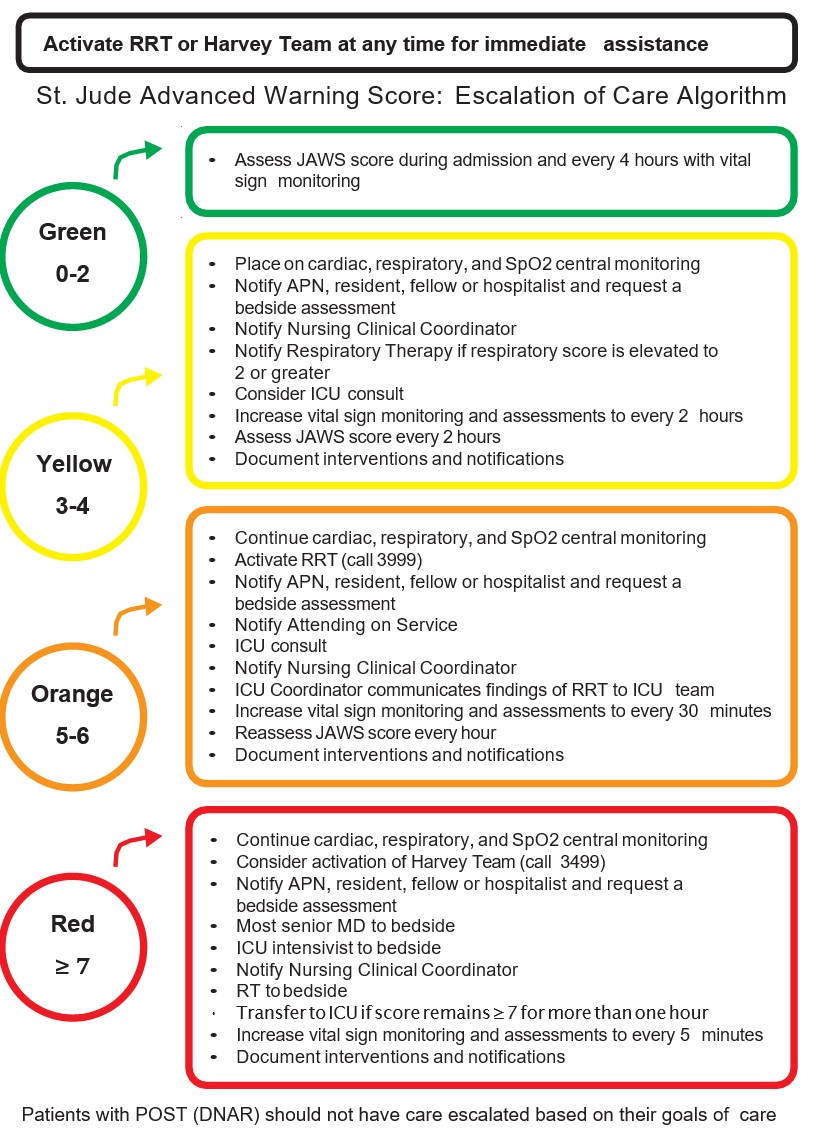
PEWS escalation algorithm from St. Jude Children’s Research Hospital (SJCRH). Reproduced with permission from Graetz D, Kaye EC, Garza M, Ferrara G, Rodriguez M, Soberanis Vásquez DJ, Méndez Aceituno A, Antillon-Klussmann F, Gattuso JS, Mandrell BN, Baker JN, Rodriguez-Galindo C, Mack JW, Agulnik A. Qualitative Study of Pediatric Early Warning Systems' Impact on Interdisciplinary Communication in Two Pediatric Oncology Hospitals With Varying Resources. JCO Glob Oncol. 2020 Jul;6:1079-108
